# Seed dormancy-life form profile for 358 species from the Xishuangbanna seasonal tropical rainforest, Yunnan Province, China compared to world database

**DOI:** 10.1038/s41598-018-22930-5

**Published:** 2018-03-16

**Authors:** Qinying Lan, Shouhua Yin, Huiyin He, Yunhong Tan, Qiang Liu, Yongmei Xia, Bin Wen, Carol C. Baskin, Jerry M. Baskin

**Affiliations:** 1 Center for Integrative Conservation, Xishuangbanna Tropical Botanical Garden, Chinese Academy of Sciences, Germplasm Bank, Mengla, 666303 Yunnan, China; 2 Department of Biology, University of Kentucky, Lexington, Kentucky 40506 USA; 3 Department of Plant and Soil Sciences, University of Kentucky, Lexington, Kentucky 40546 USA

## Abstract

Seed dormancy profiles are available for the major vegetation regions/types on earth. These were constructed using a composite of data from locations within each region. Furthermore, the proportion of species with nondormant (ND) seeds and the five classes of dormancy is available for each life form in each region. Using these data, we asked: will the results be the same if many species from a specific area as opposed to data compiled from many locationsare considered? Germination was tested for fresh seeds of 358 species in 95 families from the Xishuangbanna seasonal tropical rainforest (XSTRF): 177 trees, 66 shrubs, 57 vines and 58 herbs. Seeds of 12.3% of the species were ND, and 0.3, 14.8, 60.6, 12.0 and 0% of the species had morphological (MD), morphophysiological (MPD), physiological (PD), physical (PY), and combinational (PY + PD) dormancy, respectively. PD was more important than ND in all life forms, PY was highest in shrubs, MD was not important in any life form and MPD was most common for herb and vines. The seed dormancy profile for XSTRF differs considerably from the composite profile for this vegetation type worldwide, most obviously in ND being much lower and PD much higher in XSTRF.

## Introduction

Timing of seed germination in nature can be viewed as an adaptation of a species to its habitat. That is, unless environmental conditions are favorable for seedling establishment at the time seeds germinate the seedlings will die. Further, even if seedlings can survive, germination at some times during the germination season may be more favorable for growth than at others. Consequently, as many studies have shown, timing of germination can have a major effect not only on seedling survival but also on lifetime fitness of the resulting plants, especially annuals^1^. For many species, seed dormancy, including when and how it is broken, is an important aspect of how the timing of seed germination is controlled in the field.

If we wish to understand the timing of germination in the major vegetation regions on earth, information is needed on the relative proportion of species with dormant and nondormant seeds in each region. Furthermore, we need to know the proportion of the different classes of seed dormancy^2^: morphological (MD), morphopysiological (MPD), physiological (PD), physical (PY) and combinational (PY + PD). When information about seed dormancy is obtained for a vegetation type in a particular region, a dormancy profile can be constructed, showing the relative importance of nondormancy (ND) and of MD, MPD, PD, PY and PY + PD. Dormancy profiles for all the major vegetation regions on earth allow us to compare the importance of each class of dormancy (and ND) across broad environmental gradients. Such an approach permits an evaluation of the role of seed dormancy/nondormancy in the adaptation of species to different climatic zones. In addition, for each vegetation region we can evaluate the seed dormancy profile for each life form.

A world biogeography of seed dormancy (and ND) was constructed by Baskin and Baskin^1^, using information for 13, 634 species, and this information has been used to compare life forms and seed dormancy in the various vegetation regions worldwide^1^. However, for each vegetation region, e.g. the semi-evergreen (seasonal) tropical rainforest, seed dormancy and dormancy-life form profiles are based on a composite of information generated from studies conducted at many locations within the vegetation type. Thus, we asked: would the seed dormancy-life form profiles be the same if data from a specific area as opposed to data compiled from many locations in the same type of vegetation in various parts of the world are considered??

To begin to try to answer this question, seed dormancy studies were conducted on seeds of 182 trees, 65 shrubs, 58 vines and 55 herbs growing in the semi-evergreen (seasonal) tropical rainforest of southern Yunnan Province, China. Based on data compiled for seasonal tropical rainforests from various parts of the world, we predicted that (1) 40–45% of the species have ND seeds; and (2) ND and PD are about equally important in each life form, MD and MPD are not important in any life form and PY is more important in herbs than in other life forms. To test these hypotheses, we asked two specific questions. (1) What is the proportion of species with ND seeds and with each of the five classes of dormancy? (2) Is occurrence of ND and of the different classes of dormancy correlated with life form?

## Results

### Dormancy profile

**Figure 1:**
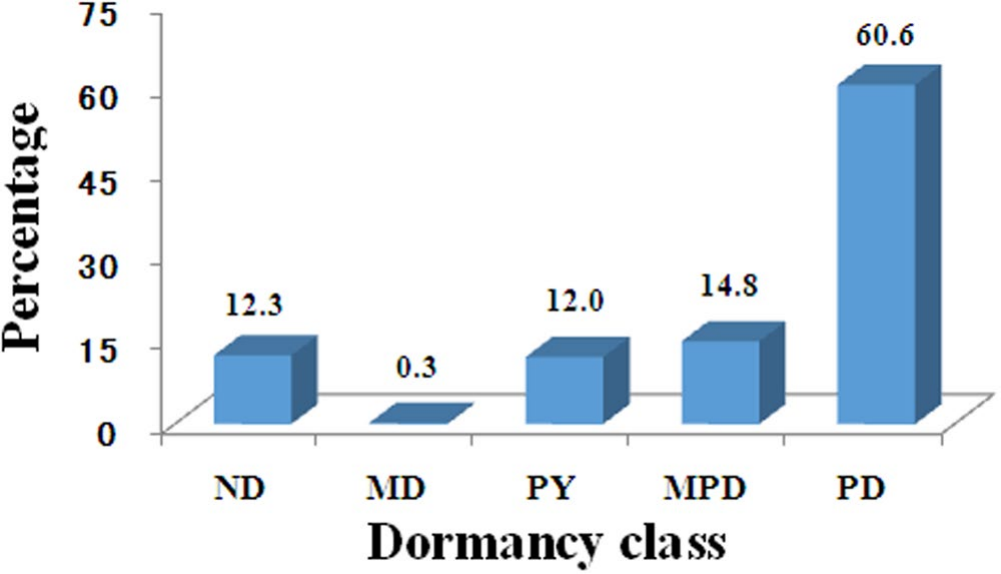
Seed dormancy profile for 358 species from the Xishuangbanna seasonal tropical rainforest in southern Yunnan Province, China. ND, nondormant; MD, morphological dormancy; MPD, morphophysiological dormancy; PD, physiological dormancy; PY, physical dormancy.

Seed germination data were collected for 358 species/taxa in 95 families from the Xishaungbanna seasonal tropical rainforest (Table S1). Seeds of 44 species in 19 families were ND (12.3%), while 314 species in 93 families were dormant (87.7%) (Fig. 1). Seed dormancy class depended on the family to which the species belonged (X-square = 437.7222, df = 4, p < 0.001). Within some families, seeds of some species were ND, while in other species they were dormant, e.g. Moraceae, 13 species ND and 6 species dormant; Myrsinaceae, 1 ND and 9 dormant; and Rubiaceae, 1 species ND and 17 dormant. In the Lauraceae, all 15 species tested had dormant seeds. PD was present in 217 of the 358 species (60.6%) in 62 families. The families with 7 or more species whose seeds had PD were Cucurbitaceae, 13 species; Euphorbiaceae, 23; Lauraceae, 15; Myrsinaceae, 9; Rubiaceae, 16; Verbenaceae, 7; and Zingiberaceae, 7.

PY occurred in 43 species in 8 families; the most important of which were Fabaceae, 24 species and Malvaceae, 5. MPD was recorded in 53 species in 23 families, the most important of which were Vitaceae, 11 species, Araliaceae, 6; Arecaceae, 4; Aquifoliaceae, 3; Myrsticaceae, 3; and Pittosporaceae, 3. MD occurred only in *Musa acuminata* (Musaceae).

### Seed dormancy-life form

**Figure 2:**
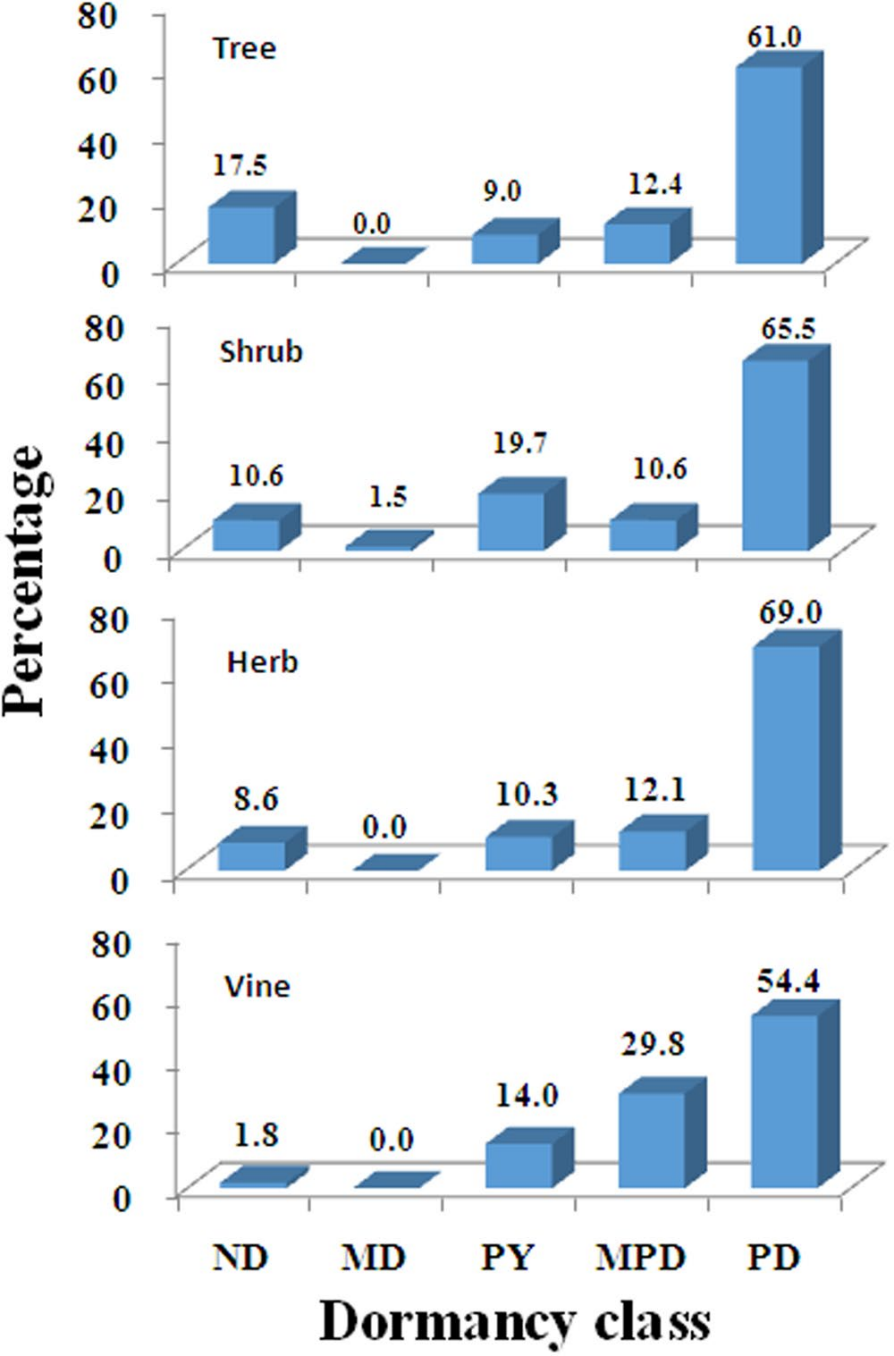
Seed dormancy profile for the four plant life forms in the Xishuangbanna seasonal tropical rainforest. ND, nondormant; MD, morphological dormancy; MPD, morphophysiological dormancy; PD, physiological dormancy; PY, physical dormancy.

For the most part, each of the five classes of dormancy was fairly evenly distributed across growth forms (trees, shrubs, lianas and herbs; X-square = 15.08572, df = 12, p = 0.2367819). Some species within all life forms had ND seeds, but trees had the highest number of species with ND seeds (Fig. 2). MD was not important for any life form, and MPD was most common for vines and herb. PD was very common (54.4 to 69%) in all life forms, while PY accounted for 9 to 19.7% of the species with each life form.

## Discussion

Our prediction that 40–45% of the species in the Xishuangbanna seasonal tropical rainforest have ND seeds was not supported, since only 12.3% of the species had ND seeds (Fig. 1). In the data compiled by Baskin and Baskin^1^ for semi-evergreen (seasonal) tropical rainforests from various parts of the world, 42.7, 1.9, 6.2, 34.8, 14.3 and 0.07% of the species had ND, MD, MPD, PD, PY and PY + PD, respectively^1^, whereas for Xishuangbanna it was 12.3, 0.3, 14.8, 60.6, 12.0 and 0%, respectively (Fig. 1). Thus, the most obvious differences for the Xishuangbanna species compared to the world data set for the seasonal tropical rainforest are a 30.4% decrease in proportion of ND and a 26.7% increase in proportion of PD.

One reason for the differences in relative importance of ND and PD in the two data sets is that the world data set for seasonal tropical rainforests contains a higher proportion of trees with ND seeds than the Xishuangbanna data set. In the world data set, 1122 of 1430 species (78.5%) were trees, and 45.4 and 34.5% of them had ND and PD seeds, respectively^1^. In the Xishuangbanna data set, 177 of the 358 species (49.4%) were trees, and 17.5 and 61.0 had ND and PD seeds, respectively. Since 45.4% of the trees in the world data set for seasonal tropical rainforests had ND seeds^1^, we have to ask if increasing the relatively low proportion of trees in the Xishuangbanna data set is responsible for this difference. The presence of a high percentage of species, trees in particular, in tropical rainforests with ND seeds^1^ agrees very well with the continuously warm and wet, or at least seasonally wet, conditions in these forests. Under warm, wet conditions, there would be relatively low selective advantage for dormancy to evolve. In fact, given the high amount of ND that has evolved in lineages with PD^3^, it appears that these lineages have given rise to many species whose seeds are ND, supposedly because there was no selective advantage for the seeds to be dormant in various wet uniform habitats.

Our prediction regarding the relative importance of ND and the different classes of dormancy in trees, shrubs, vines and herbs also was not supported. ND and PD were not equally important in any life form, with ND ranging from 1.8 to 17.5% and PD from 54.4 to 69% in the four life forms (Fig. 2), i.e. in all life forms, the importance of PD was much higher than that of ND. In the world data set, ND ranged from 25.5 to 45.5% and PD from 32.8 to 41.5% in all life forms. As predicted, MD was not important (0–0.3%) for any life form. However, MPD was present in seeds of 29.8% of the Xishuangbanna vine species, including 11 species of Vitaceae. In the world data set, MPD in the tropical seasonal rainforest ranged from 1.4 to 9.8%, regardless of life form^1^.

In the Xishuangbanna seasonal tropical forest, 9, 19.7, 14 and 10.3% of the trees, shrubs, vines and herbs, respectively, had seeds with PY. Thus, contrary to our prediction that PY would be highest in Xishuangbanna herbs, it was highest in shrubs. In the world data set, seeds of 11.8, 12.3, 18.0 and 34.0% of seasonal tropical forest trees, shrubs, vines and herbs, respectively, had PY^1^.

One of the most obvious results from our study is that the dormancy profile is similar for each life form, with the exceptions of a relatively high value for ND in trees and the relatively high value for MPD in herb and vines (Fig. 2). Perhaps the similarity of the seed dormancy profile for the different life forms is related to the fact that regardless of life form all the species are exposed to the same environmental factors in the Xishuangbanna tropical forest.

Now, we return to our original question. Is a seed dormancy or seed dormancy-life form profile based on a large number of species from a specific area the same as the one constructed using data from sites throughout the range of the type of vegetation? In the case of the Xishuangbanna seasonal tropical rainforest, the answer generally is “no.” However, the greater importance of PD than of ND and of other classes of dormancy is true for both the Xishuangbanna species and the world data set for seasonal tropical rainforest. The overall importance of PD among the Xishuangbanna species is not surprising, since it is the most common class of dormancy in all vegetation zones on earth^1^. Further, analysis of the evolutionary transitions between dormancy classes (including ND) has shown that PD has functioned as a hub in the evolution of the various classes of seed dormancy^3^.

## Materials and Methods

### Study site

The seasonal tropical rainforest in this study is located in the Xishuangbanna Dai Autonomous Prefecture of southern Yunnan Province, China^4^. The forest is biogeographically located in a transitional zone between the tropical evergreen rainforests of southeastern Asia and the broadleaved evergreen (subtropical) forest of southwestern China. Southern Yunnan occurs at the junction of the Indian and Burmese plates of Gondwana and the Eurasian plate of Laurasia^5^.

The region has a typical monsoon climate with a dry season from November to April and a rainy season from May and October, when 84% of the annual precipitation of 1,221 mm occurs. However, foggy days during the dry season increase the humidity and help compensate for the low rainfall^6^. Mean annual temperature varies from 21.7 °C at an elevation of 550 m to 15.1 °C at 1,979 m, and the 20 °C isotherm is the same as the 850 m elevation isoline^5^.

### Seed collecting and germination tests

Seeds were collected from five or more plants growing in the forest in August to October in 1997 to 2005. Ripe seeds were collected at the beginning of the natural dispersal period. Within 3–7 days after collection, seeds were transported to the laboratory, where they were cleaned, air dried and tested for germination.

For germination tests, four replicates of 10~100 seeds each were sown on 1% agar in Petri dishes and incubated in temperature-controlled incubators at 20, 25 and 30 °C, with 14 h light (20 μmol/m^2^·s provided by cool white fluorescent tubes)/10 h dark per day for 1–2 months. Seeds were checked for germination at weekly-intervals, and the criterion for germination was protrusion of the radical to a length of 2 mm.

### Definition of seed dormancy

First, we divided the species into two categories: nondormant (ND) and dormant. Seeds were considered to be ND if they had a fully developed embryo and germinated to a high percentage over the range of test conditions in about 30 days or less.

Following Baskin and Baskin^2^, the class of dormancy was assigned to each species with dormant seeds: (1) physical dormancy (PY), a water impermeable seed (or fruit) coat (with a fully developed, nondormant embryo); (2) morphological dormancy (MD), an underdeveloped embryo, i.e. small embryo must grow inside the seed before germination can occur^7^, and embryo growth and germination occur in about 30 days or less; (3) physiological dormancy (PD), a water-permeable seed (or fruit) coat, a fully developed embryo that has low growth potential and seeds require more than 30 days to germination; (4) morphophysiological dormancy (MPD), seeds have an underdeveloped embryo that is physiologically dormant; and (5) combinational dormancy (PY + PD), seeds have a water-impermeable seed (or fruit) coat and the embryo has physiological dormancy.

If seeds were dormant and belonged to a family known to have water-impermeable seed/fruit coats (see Table 3.14 in Baskin and Baskin^1^), they were listed as having PY. If a species belonged to a family known to have underdeveloped embryos (see Table 3.11 in Baskin and Baskin^1^) and seeds germinated in <30 days, it was listed as having MD. If the species belonged to a family known to have underdeveloped embryos and seeds required >30 days to germinate, it was listed as having MPD. If the species belonged to a family not known to have PY or underdeveloped embryos and seeds required <30 days to germinate, it was listed as having ND seeds. If the species belonged to a family not known to have PY or underdeveloped embryos and seeds required >30 days to germinate, it was listed as having PD. In addition, the world data base compiled for studies on individual species in the seasonal tropical rainforest was consulted for information on each of the 358 species. Finally, no species was listed as having PY + PD because none belonged to a family with PY and had been listed by other authors as having this class of dormancy.

### Analysis

We compared the number of species with ND, MD, MPD, PD and PY, i.e. df = 4, using the chi-square test. For the most part, ND, MD, MPD, PD and PY were fairly evenly distributed across growth forms (trees, shrubs, lianas and herbs). The chi-square test was performed using R software (Version R i386 3.0.1).The differences were considered significant when the P value was less than 0.05.

## Electronic supplementary material

Supplementary file

## References

[1] Baskin, C. C. & Baskin, J. M. Seeds: Ecology, Biogeography, and Evolution of Dormancy and Germination. (Academic Press, 2014).

[2] Baskin JM, Baskin CC (2004). A classification system for seed dormancy. Seed Sci. Res..

[3] Willis C (2014). Rubio de Casas, R. The evolution of seed dormancy: environmental cues, evolutionary hubs, and diversification of the seed plants. New Phytol..

[4] Cao, M., *et al* Xishuangbanna tropical seasonal rainforest dynamics plot: tree distribution maps, diameter tables and species documentation. (Science and Technology Press, 2008).

[5] Zhu H, Cao M, Hu HB (2006). Geological history, flora, and vegetation of Xishuangbanna, Southern Yunnan, China. Biotropic.

[6] Zhang JH, Cao M (1995). Tropical forest vegetation of Xishuangbanna, SW China and its secondary changes, with special reference to some problems in local nature conservation. Biol. Conserv..

[7] Grushvitzky, I. V. After-ripening of seeds of primitive tribes of angiosperms, conditions and peculiarities. Vol. 1. (ed. Borriss, H.) 329–336. (University of Griefswald, 1967).

