# Supplementary material for: Seed dormancy-life form profile for 358 species from the Xishuangbanna seasonal tropical rainforest, Yunnan Province, China compared to world database
Source: Sci Rep. 2018 Mar 16;8:4674. doi: 10.1038/s41598-018-22930-5 (PMC5856844; doi:10.1038/s41598-018-22930-5)
Supplement: Supplementary file 1 — Supplementary file [file 41598_2018_22930_MOESM1_ESM.doc]

**Seed dormancy-life form profile for 358 species from the Xishuangbanna seasonal tropical rainforest, Yunnan Province, China compared to world database**

Qinying Lan^1^, Shouhua Yin^1^, Huiyin He^1^, Yunhong Tan^1^ , Qiang Liu^1^, Yongmei Xia^1^, Bin Wen^1^,Carol C. Baskin^2^,^3^ , Jerry M. Baskin^2^

^1^Center for Integrative Conservation, Xishuangbanna Tropical Botanical Garden, Chinese Academy of Sciences, Germplasm Bank, Mengla 666303, Yunnan, China. ^2^Department of Biology, University of Kentucky, Lexington, Kentucky 40506 USA; and ^3^Department of Plant and Soil Sciences, University of Kentucky, Lexington, Kentucky 40546 USA. Correspondence and requests for materials should be addressed to C.C.B. ([carol.baskin@uky.edu](mailto:carol.baskin@uky.edu))

| **Table S1. Germination percentages after 1 and 2 months of incubation at three temperatures of seeds of 358 species of trees, shrubs, vines and herbscollected from the Xishuanbanna semi-evergreen (seasonal)** | | | | | | | | | |
| --- | --- | --- | --- | --- | --- | --- | --- | --- | --- |
| **tropical forest in Yunnan Province, southwestern China** | | | | | | | | |  |
|  | | | | | | | | | |
| **Family** | **Species** | **Life** | **Germination percentage** | | | **Germination percentage** | | | **Kinds of** |
|  |  |  | **(1 month)** | | | **(2 month)** | | | **dormancy** |
|  |  |  | **20℃** | **25℃** | **30℃** | **20℃** | **25℃** | **30℃** |  |
| **Actinidiaceae** | **Saurauia tristyla DC.** | **tree** | **85** | **88** | **87** | **87** | **91** | **92** | **ND** |
| **Alangiaceae** | **Alangium chinense (Lour.) Harms** | **tree** | **0** | **0** | **0** | **0** | **0** | **0** | **PD** |
| **Alangiaceae** | **Alangium kurzii Craib var. kurzii** | **tree** | **0** | **0** | **0** | **0** | **0** | **0** | **PD** |
| **Anacardiaceae** | **Choerospondias axillaris (Roxb.) B. L. Burtt & A. W. Hill** | **tree** | **0** | **0** | **0** | **0** | **0** | **0** | **PD** |
| **Anacardiaceae** | **Fordia cauliflora Hemsl.** | **shrub** | **0** | **0** | **0** | **0** | **0** | **1** | **PD** |
| **Anacardiaceae** | **Rhus chinensis Mill.** | **tree** | **14** | **21** | **16** | **28** | **55** | **21** | **PY** |
| **Anacardiaceae** | **Toxicodendron acuminatum (DC.) C. Y. Wu et T. L. Ming** | **tree** | **3** | **33** | **16** | **95** | **87** | **77** | **PY** |
| **Anacardiaceae** | **Toxicodendron succedaneum (L.) O. Kuntze** | **tree** | **0** | **6** | **4** | **56** | **10** | **78** | **PY** |
| **Annonaceae** | **Fissistigma wallichii (Hook. f. et Thoms.) Merr.** | **vine** | **0** | **0** | **0** | **0** | **0** | **0** | **MPD** |
| **Annonaceae** | **Goniothalamus griffithii Hook. f. et Thoms.** | **tree** | **0** | **0** | **0** | **0** | **9** | **2** | **MPD** |
| **Annonaceae** | **Polyalthia cheliensis Hu** | **tree** | **80** | **47** | **47** | **90** | **80** | **90** | **MPD** |
| **Apocynaceae** | **Ervatamia mucronata (Merr.) Markgr.** | **shrub** | **0** | **0** | **0** | **0** | **0** | **0** | **PD** |
| **Apocynaceae** | **Rauvolfia yunnanensis Tsiang** | **shrub** | **0** | **0** | **0** | **0** | **0** | **0** | **PD** |
| **Apocynaceae** | **Tabernaemontana divaricata L.** | **shrub** | **0** | **0** | **0** | **0** | **0** | **0** | **PD** |
| **Aquifoliaceae** | **Ilex corallina Franch.** | **tree** | **0** | **0** | **0** | **0** | **0** | **0** | **MPD** |
| **Aquifoliaceae** | **Ilex micrococca Maxim.** | **tree** | **0** | **0** | **0** | **0** | **0** | **0** | **MPD** |
| **Aquifoliaceae** | **Ilex polyneura (Hand.-Mazz.) S. Y. Hu** | **tree** | **0** | **0** | **0** | **0** | **0** | **0** | **MPD** |
| **Araceae** | **Amorphophallus virosus N. E. Brown** | **herb** | **0** | **0** | **0** | **0** | **0** | **0** | **PD** |
| **Araceae** | **Pothos chinensis (Raf.) Merr.** | **herb** | **13** | **24** | **13** | **24** | **29** | **31** | **PD** |
| **Araliaceae** | **Aralia armata(Wall.)Seem.** | **shrub** | **0** | **7** | **0** | **0** | **9** | **0** | **MPD** |
| **Araliaceae** | **Colona floribunda (Wall.) Craib** | **tree** | **0** | **0** | **0** | **0** | **0** | **0** | **MPD** |
| **Araliaceae** | **Heteropanax fragrans (Roxb.) Seem.** | **tree** | **0** | **0** | **0** | **0** | **0** | **0** | **MPD** |
| **Araliaceae** | **Schefflera octophylla (Lour.) Harms** | **shrub** | **0** | **0** | **0** | **0** | **0** | **0** | **MPD** |
| **Araliaceae** | **Schefflera venulosa (Wight et Arn.) Harms** | **tree** | **0** | **0** | **0** | **0** | **0** | **0** | **MPD** |
| **Araliaceae** | **Trevesia palmata (Roxb.) Vis.** | **tree** | **0** | **0** | **0** | **0** | **0** | **0** | **MPD** |
| **Arecaceae** | **Calamus nambariensis Becc.** | **vine** | **0** | **0** | **0** | **0** | **0** | **0** | **MPD** |
| **Arecaceae** | **Caryota urens L.** | **herb** | **0** | **0** | **0** | **25** | **10** | **15** | **MPD** |
| **Arecaceae** | **Caryota ochlandra Hance** | **herb** | **0** | **2** | **0** | **25** | **42** | **47** | **MPD** |
| **Arecaceae** | **Trachycarpus fortunei (Hook.) H. Wendl.** | **herb** | **18** | **22** | **8** | **33** | **37** | **37** | **MPD** |
| **Asteraceae** | **Eupatorium fortunei Turcz.** | **herb** | **0** | **0** | **0** | **100** | **90** | **72** | **PD** |
| **Bignoniaceae** | **Mayodendron igneum (Kurz.) Kurz.** | **tree** | **0** | **0** | **0** | **0** | **18** | **18** | **PD** |
| **Bixaceae** | **Bixa orellana L.** | **tree** | **9** | **12** | **19** | **24** | **17** | **19** | **PY** |
| **Boraginaceae** | **Canarium pimela Leenh.** | **tree** | **0** | **10** | **3** | **77** | **63** | **57** | **PD** |
| **Boraginaceae** | **Cordia dichotoma Forst.** | **tree** | **0** | **0** | **0** | **0** | **0** | **17** | **PD** |
| **Boraginaceae** | **Ehretia acuminata R.Br** | **tree** | **0** | **3** | **0** | **0** | **6** | **6** | **PD** |
| **Boraginaceae** | **Ehretia thyrsiflora (Sieb. et Zucc.) Nakai** | **tree** | **0** | **0** | **0** | **0** | **0** | **0** | **PD** |
| **Buddlejaceae** | **Buddleja asiatica Lour.** | **tree** | **0** | **0** | **/** | **99** | **99** | **/** | **PD** |
| **Burseraceae** | **Canarium strictum Roxb.** | **tree** | **0** | **0** | **0** | **3** | **33** | **47** | **PD** |
| **Caprifoliaceae** | **Sambucus chinensis Lindl.** | **herb** | **0** | **0** | **0** | **0** | **0** | **0** | **MPD** |
| **Cardiopteridaceae** | **Peripterygium platycarpum (Gagn.) Sleum.** | **vine** | **0** | **0** | **0** | **0** | **0** | **0** | **PD** |
| **Cardiopteridaceae** | **Peripterygium quinquelobum Hassk.** | **vine** | **0** | **0** | **0** | **0** | **0** | **0** | **PD** |
| **Celastraceae** | **Celastrus paniculatus Willd.** | **tree** | **0** | **0** | **0** | **0** | **0** | **0** | **PD** |
| **Celastraceae** | **Celastrus sp.** | **tree** | **50** | **50** | **65** | **98** | **100** | **88** | **PD** |
| **Celastraceae** | **Celastrus monospermus Roxb.** | **vine** | **22** | **20** | **42** | **82** | **87** | **42** | **PD** |
| **Cephalotaxaceae** | **Cephalotaxus mannii Hook. f.** | **tree** | **0** | **0** | **0** | **43** | **83** | **0** | **MPD** |
| **Chloranthaceae** | **Sarcandra hainanensis (Pei) Swamy et Bail.** | **shrub** | **0** | **0** | **7** | **64** | **69** | **9** | **MPD** |
| **Combretaceae** | **Anogeissus var. lanceolata Wall. ex C. B. Clarke** | **tree** | **0** | **0** | **0** | **0** | **0** | **0** | **PD** |
| **Combretaceae** | **Terminalia argyrophylla Pott. et Prain** | **tree** | **0** | **0** | **0** | **0** | **24** | **24** | **PD** |
| **Combretaceae** | **Terminalia bellirica (Faertn.)Roxb.** | **tree** | **0** | **0** | **0** | **0** | **16** | **16** | **PD** |
| **Combretaceae** | **Terminalia chebula Retz.** | **tree** | **0** | **0** | **0** | **0** | **0** | **0** | **PD** |
| **Combretaceae** | **Terminalia myriocarpa Van Huerck** | **tree** | **85** | **89** | **85** | **90** | **89** | **90** | **ND** |
| **Commelinaceae** | **Pollia subumbellata C. B. Clarke** | **herb** | **0** | **0** | **0** | **0** | **47** | **0** | **PD** |
| **Convolvuaceae** | **Merremia vitifolia (Burm. f.) Hall. f.** | **vine** | **18** | **37** | **14** | **30** | **44** | **58** | **PY** |
| **Convolvuaceae** | **Merremia sp.** | **vine** | **4** | **5** | **4** | **8** | **7** | **6** | **PY** |
| **Convolvuaceae** | **Porana henryi Verdc.** | **herb** | **43** | **38** | **38** | **48** | **54** | **58** | **PY** |
| **Cucurbitaceae** | **Cucumis melo L.** | **herb** | **0** | **0** | **0** | **0** | **0** | **0** | **PD** |
| **Cucurbitaceae** | **Gynostemma burmanicum King ex Chakr.** | **vine** | **0** | **0** | **0** | **0** | **17** | **43** | **PD** |
| **Cucurbitaceae** | **Gynostemma pentaphyllum (Thunb.) Makino** | **herb** | **32** | **19** | **32** | **76** | **54** | **87** | **PD** |
| **Cucurbitaceae** | **Momordica cochinchinensis (Lour.) Spreng.** | **vine** | **0** | **0** | **0** | **0** | **0** | **2** | **PD** |
| **Cucurbitaceae** | **Thladiantha cordifolia (Bl.) Cogn. var. tomentosa A.M.Lu et Z.Y.Zhang** | **herb** | **39** | **19** | **20** | **41** | **42** | **41** | **PD** |
| **Cucurbitaceae** | **Thladiantha villosula Cogn.** | **vine** | **0** | **0** | **0** | **0** | **0** | **0** | **PD** |
| **Cucurbitaceae** | **Trichosanthes kirilowii Maxim.** | **herb** | **0** | **94** | **86** | **86** | **98** | **98** | **ND** |
| **Cucurbitaceae** | **Trichosanthes quinquangulata A. Gray** | **herb** | **0** | **0** | **10** | **3** | **3** | **10** | **PD** |
| **Cucurbitaceae** | **Trichosanthes rubriflos Thorel ex Cayla** | **vine** | **0** | **0** | **0** | **0** | **40** | **44** | **PD** |
| **Cucurbitaceae** | **Trichosanthes sp.** | **herb** | **0** | **30** | **0** | **0** | **57** | **0** | **PD** |
| **Cucurbitaceae** | **Trichosanthes sp.** | **vine** | **0** | **0** | **0** | **0** | **0** | **7** | **PD** |
| **Cucurbitaceae** | **Trichosanthes sp.** | **vine** | **0** | **0** | **0** | **0** | **0** | **0** | **PD** |
| **Cucurbitaceae** | **Trichosanthes villosa Bl.** | **herb** | **0** | **0** | **0** | **0** | **0** | **0** | **PD** |
| **Cucurbitaceae** | **Zehneria sp.** | **herb** | **47** | **47** | **63** | **63** | **73** | **73** | **PD** |
| **Cucurbitaceae** | **Zehneria indica (Lour.) Keraudren** | **herb** | **98** | **46** | **46** | **99** | **99** | **98** | **ND** |
| **Cycadaceae** | **Cycas pectinata Griff.** | **tree** | **0** | **0** | **0** | **0** | **0** | **0** | **MPD** |
| **Cyperaceae** | **Carex baccans Nees** | **herb** | **0** | **0** | **5** | **27** | **52** | **15** | **PD** |
| **Cyperaceae** | **Carex sp.** | **herb** | **1** | **4** | **1** | **50** | **43** | **49** | **PD** |
| **Daphniphyllaceae** | **Daphniphyllum paxianum K. Rosenthal** | **shrub** | **0** | **1** | **0** | **69** | **87** | **36** | **MPD** |
| **Dioscoreaceae** | **Dioscorea sp.** | **vine** | **5** | **8** | **8** | **41** | **8** | **62** | **PD** |
| **Dioscoreaceae** | **Dioscorea sp.** | **vine** | **7** | **13** | **16** | **44** | **25** | **23** | **PD** |
| **Dipterocarpaceae** | **Dipterocarpus tubinatus Gaertn.f.** | **tree** | **90** | **90** | **100** | **90** | **90** | **100** | **ND** |
| **Dipterocarpaceae** | **Parashorea chinensis Wang Hsie** | **tree** | **87** | **87** | **100** | **100** | **100** | **100** | **ND** |
| **Ebenaceae** | **Diospyros morrisiana Hance** | **tree** | **0** | **0** | **0** | **67** | **40** | **57** | **PD** |
| **Elaeocarpaceae** | **Elaeocarpus petiolatus (Jack) Wall. ex Kurz** | **tree** | **0** | **0** | **0** | **0** | **0** | **0** | **PD** |
| **Elaeocarpaceae** | **Elaeocarpus varunua Buch.-Ham.** | **tree** | **0** | **0** | **0** | **0** | **0** | **0** | **PD** |
| **Ericaceae** | **Agapetes mannii Hemsl.** | **shrub** | **0** | **17** | **0** | **0** | **17** | **0** | **PD** |
| **Ericaceae** | **Vaccinium bracteatum Thunb.** | **shrub** | **0** | **0** | **0** | **67** | **66** | **75** | **PD** |
| **Euphorbiaceae** | **Antidesma venosum E. Mey. ex Tul.** | **shrub** | **0** | **0** | **3** | **40** | **18** | **25** | **PD** |
| **Euphorbiaceae** | **Antidesma bunius (L.) Spreng.** | **tree** | **0** | **0** | **0** | **17** | **31** | **13** | **PD** |
| **Euphorbiaceae** | **Antidesma acidum Retz** | **tree** | **0** | **0** | **0** | **20** | **15** | **9** | **PD** |
| **Euphorbiaceae** | **Aporusa octandra (Buch.-Ham.ex D. Don)Vick** | **tree** | **77** | **75** | **75** | **78** | **78** | **78** | **ND** |
| **Euphorbiaceae** | **Baliospermum montanum (Willd.) Muell. Arg.** | **tree** | **0** | **0** | **0** | **0** | **28** | **29** | **PD** |
| **Euphorbiaceae** | **Bischofia polycarpa (Levl.) Airy Shaw** | **tree** | **70** | **78** | **74** | **78** | **94** | **84** | **ND** |
| **Euphorbiaceae** | **Bridelia insulana Hance** | **shrub** | **0** | **0** | **0** | **0** | **0** | **0** | **PD** |
| **Euphorbiaceae** | **Bridelia tomentosa Bl.** | **tree** | **0** | **0** | **0** | **0** | **0** | **0** | **PD** |
| **Euphorbiaceae** | **Cleidiocarpon cavaleriei (Levl.) Airy Shaw** | **tree** | **90** | **95** | **98** | **90** | **95** | **98** | **ND** |
| **Euphorbiaceae** | **Cleidion brevipetiolatum Pax et Hoffm.** | **tree** | **0** | **0** | **2** | **0** | **0** | **2** | **PD** |
| **Euphorbiaceae** | **Cleistanthus sumatranus (Miq.) Muell. Arg.** | **tree** | **11** | **4** | **4** | **20** | **11** | **20** | **PD** |
| **Euphorbiaceae** | **Claoxylon longifolium (Bl.) Endl. ex Hassk.** | **tree** | **0** | **0** | **0** | **0** | **0** | **0** | **PD** |
| **Euphorbiaceae** | **Croton kongensis Gagnep.** | **shrub** | **0** | **0** | **0** | **0** | **0** | **0** | **PD** |
| **Euphorbiaceae** | **Flueggea virosa (Roxb. ex Willd.) Voigt** | **tree** | **0** | **0** | **0** | **14** | **14** | **0** | **PD** |
| **Euphorbiaceae** | **Homonoia riparia Lour.** | **tree** | **0** | **0** | **0** | **0** | **11** | **11** | **PD** |
| **Euphorbiaceae** | **Macaranga denticulata (Bl.) Muell. Arg.** | **tree** | **0** | **0** | **0** | **0** | **10** | **17** | **PD** |
| **Euphorbiaceae** | **Macaranga indica Wight** | **tree** | **0** | **0** | **0** | **0** | **7** | **7** | **PD** |
| **Euphorbiaceae** | **Macaranga kurzii (Kuntze) Pax et Hoffm.** | **shrub** | **0** | **0** | **0** | **0** | **1** | **1** | **PD** |
| **Euphorbiaceae** | **Macaranga tanarius (L.) Muell. Arg.** | **tree** | **0** | **0** | **1** | **1** | **4** | **4** | **PD** |
| **Euphorbiaceae** | **Mallotus apelta (Lour.) Muell. Arg.** | **tree** | **0** | **0** | **1** | **1** | **3** | **3** | **PD** |
| **Euphorbiaceae** | **Mallotus barbatus (Wall.) Muell. Arg.** | **shrub** | **0** | **0** | **0** | **31** | **8** | **90** | **PD** |
| **Euphorbiaceae** | **Mallotus macrostachys (Miq.)Muell.-Arg.** | **tree** | **0** | **0** | **0** | **68** | **35** | **49** | **PD** |
| **Euphorbiaceae** | **Mallotus paniculatus (Lam.) Muell. Arg.** | **tree** | **0** | **0** | **0** | **0** | **0** | **0** | **PD** |
| **Euphorbiaceae** | **Ostodes paniculata Bl.** | **tree** | **10** | **13** | **17** | **17** | **20** | **20** | **PD** |
| **Euphorbiaceae** | **Phyllanthus emblica L.** | **tree** | **11** | **11** | **7** | **39** | **12** | **12** | **PD** |
| **Euphorbiaceae** | **Sumbaviopsis albicans (Bl.) J. J. Smith** | **tree** | **18** | **27** | **22** | **22** | **38** | **38** | **PD** |
| **Fabaceae** | **Acacia tonkinensis I.C.Nielsen** | **vine** | **13** | **13** | **20** | **20** | **20** | **20** | **PY** |
| **Fabaceae** | **Albizia julibrissin Durazz.** | **tree** | **0** | **0** | **0** | **60** | **49** | **63** | **PY** |
| **Fabaceae** | **Albizia odoratissima (L. f.) Benth.** | **tree** | **0** | **0** | **0** | **0** | **0** | **0** | **PY** |
| **Fabaceae** | **Bauhinia glauca (Wall. ex Benth.) Benth. subsp. tenuiflora (Watt ex C.B.Clarke)K.et S.S.Lar.** | **vine** | **0** | **0** | **0** | **0** | **2** | **2** | **PY** |
| **Fabaceae** | **Caesalpinia minax Hance** | **shrub** | **3** | **7** | **17** | **17** | **7** | **17** | **PY** |
| **Fabaceae** | **Cajanus grandiflorus (Benth. ex Baker) Vaniot der Maesen** | **herb** | **13** | **13** | **33** | **42** | **18** | **53** | **PY** |
| **Fabaceae** | **Cassia sp.** | **herb** | **61** | **89** | **88** | **91** | **97** | **97** | **ND** |
| **Fabaceae** | **Cassia nodosa Buch.-Ham.** | **tree** | **16** | **11** | **11** | **29** | **31** | **33** | **PY** |
| **Fabaceae** | **Cassia occidentalis L.** | **herb** | **2** | **3** | **3** | **3** | **8** | **17** | **PY** |
| **Fabaceae** | **Codariocalyx motorius(Houtt.) Ohashi** | **herb** | **2** | **2** | **5** | **5** | **7** | **7** | **PY** |
| **Fabaceae** | **Crotalaria pallida Ait.** | **shrub** | **86** | **86** | **77** | **89** | **91** | **91** | **ND** |
| **Fabaceae** | **Crotalaria albida Heyne ex Roth** | **shrub** | **57** | **47** | **47** | **96** | **96** | **52** | **ND** |
| **Fabaceae** | **Dalbergia fusca Pierre** | **tree** | **62** | **62** | **82** | **85** | **80** | **82** | **ND** |
| **Fabaceae** | **Dalbergia sp.** | **tree** | **71** | **71** | **81** | **83** | **91** | **94** | **ND** |
| **Fabaceae** | **Dalbergia rimosa Roxb.** | **shrub** | **0** | **0** | **0** | **0** | **0** | **0** | **PY** |
| **Fabaceae** | **Desmodium sequax Wall.** | **shrub** | **0** | **0** | **0** | **30** | **33** | **38** | **PY** |
| **Fabaceae** | **Entada phaseoloides (L.) Merr.** | **vine** | **0** | **0** | **0** | **0** | **0** | **0** | **PY** |
| **Fabaceae** | **Leucaena leucocephala (Lam.) de Wit** | **tree** | **78** | **79** | **82** | **91** | **83** | **91** | **ND** |
| **Fabaceae** | **Lysidice rhodostegia Hance** | **tree** | **11** | **0** | **0** | **13** | **21** | **11** | **PY** |
| **Fabaceae** | **Pithecellobium clypearia (Jack) Benth.** | **tree** | **0** | **0** | **0** | **0** | **0** | **0** | **PY** |
| **Fabaceae** | **Pithecellobium sp.** | **tree** | **0** | **0** | **0** | **0** | **0** | **0** | **PY** |
| **Fabaceae** | **Smithia sp.** | **herb** | **94** | **91** | **93** | **96** | **96** | **94** | **ND** |
| **Fabaceae** | **Smithia sensitiva Ait.** | **herb** | **0** | **0** | **0** | **15** | **3** | **26** | **PY** |
| **Fabaceae** | **Flemingia macrophylla (Willd.) Prain** | **shrub** | **39** | **24** | **37** | **53** | **53** | **58** | **PY** |
| **Fabaceae** | **Mucuna sempervirens Hemsl.** | **vine** | **3** | **3** | **15** | **15** | **20** | **20** | **PY** |
| **Fabaceae** | **Mucuna sp.** | **vine** | **26** | **23** | **26** | **32** | **32** | **30** | **PY** |
| **Fabaceae** | **Mucuna sp.** | **vine** | **21** | **25** | **18** | **22** | **27** | **31** | **PY** |
| **Fabaceae** | **Ormosia fordiana Oliver** | **tree** | **0** | **60** | **0** | **0** | **90** | **0** | **PY** |
| **Fabaceae** | **Ormosia yunnanensis Prain** | **tree** | **0** | **0** | **2** | **0** | **0** | **2** | **PY** |
| **Fabaceae** | **Uraria crinita (L.) Desv. ex DC.** | **shrub** | **0** | **0** | **2** | **2** | **2** | **2** | **PY** |
| **Fabaceae** | **Uraria lagopodioides (L.) Desv. ex DC.** | **shrub** | **1** | **3** | **1** | **7** | **7** | **8** | **PY** |
| **Fagaceae** | **Castanea seguinii Dode** | **tree** | **7** | **0** | **0** | **17** | **63** | **27** | **PD** |
| **Fagaceae** | **Castanopsis calathiformis (Skan) Rehd. et Wils.** | **tree** | **0** | **0** | **0** | **0** | **0** | **0** | **PD** |
| **Fagaceae** | **Castanopsis hystrix J. D. Hooker et Thomson ex A. DeCandolle** | **tree** | **0** | **0** | **0** | **0** | **0** | **0** | **PD** |
| **Fagaceae** | **Lithocarpus polystachyus (Wall.) Rehd.** | **tree** | **0** | **0** | **0** | **0** | **0** | **0** | **PD** |
| **Flacourtiaceae** | **Flacourtia ramontchii L'Hér.** | **tree** | **0** | **0** | **0** | **0** | **0** | **0** | **PD** |
| **Flacourtiaceae** | **Flacourtia sp.** | **tree** | **0** | **0** | **0** | **0** | **0** | **0** | **PD** |
| **Fumariaceae** | **Dactylicapnos scandens (D. Don) Hutch.** | **herb** | **0** | **0** | **0** | **5** | **0** | **0** | **MPD** |
| **Gesneriaceae** | **Rhynchotechum obovatum (Griff.) Burtt** | **shrub** | **0** | **0** | **0** | **100** | **94** | **98** | **PD** |
| **Gnetaceae** | **Gnetum montanum Markgr. f. megalocarpum Markgr.** | **vine** | **0** | **0** | **0** | **55** | **45** | **25** | **PD** |
| **Gnetaceae** | **Gnetum montanum Markgr.** | **vine** | **0** | **0** | **0** | **21** | **4** | **30** | **PD** |
| **Gramineae** | **Coix lacryma-jobi L.** | **herb** | **0** | **10** | **10** | **0** | **13** | **48** | **PD** |
| **Gramineae** | **Echinochloa crusgalli (L.) P. Beauv.** | **herb** | **0** | **14** | **3** | **8** | **21** | **31** | **PD** |
| **Gramineae** | **Echinochloa sp.** | **herb** | **0** | **22** | **0** | **22** | **40** | **60** | **PD** |
| **Gramineae** | **Pennisetum alopecuroides (L.)Spreng.** | **herb** | **0** | **0** | **0** | **0** | **0** | **0** | **PD** |
| **Guttiferae** | **Garcinia xishuanbannaensis Y. H. Li** | **tree** | **23** | **17** | **20** | **37** | **43** | **33** | **PD** |
| **Guttiferae** | **Garcinia cowa Roxb.** | **tree** | **0** | **10** | **13** | **35** | **15** | **25** | **PD** |
| **Hydrangiaceae** | **Dichroa febrifuga Lour.** | **shrub** | **60** | **78** | **63** | **67** | **81** | **89** | **ND** |
| **Icacinaceae** | **Iodes cirrhosa Turcz.** | **vine** | **0** | **0** | **0** | **0** | **0** | **46** | **MPD** |
| **Icacinaceae** | **Mappianthus iodoides Hand.-Mazz.** | **vine** | **0** | **0** | **0** | **0** | **0** | **0** | **MPD** |
| **Iridaceae** | **Belamcanda chinensis (L.) DC.** | **herb** | **2** | **0** | **0** | **3** | **3** | **1** | **MPD** |
| **Juglandaceae** | **Engelhardia roxburghiana Wall** | **tree** | **0** | **0** | **0** | **0** | **30** | **0** | **PD** |
| **Juglandaceae** | **Engelhardia spicata Lesch.** | **tree** | **0** | **0** | **0** | **18** | **0** | **2** | **PD** |
| **Juglandaceae** | **Juglans cathayensis Dode** | **tree** | **0** | **0** | **0** | **0** | **0** | **0** | **PD** |
| **Juglandaceae** | **Juglans regia L.** | **tree** | **0** | **0** | **0** | **0** | **0** | **0** | **PD** |
| **Labiatae** | **Anisomeles indica (L.) Kuntze** | **herb** | **0** | **0** | **0** | **0** | **13** | **0** | **PD** |
| **Labiatae** | **Craniotome furcata (Link) O. Ktze.** | **herb** | **/** | **0** | **0** | **/** | **93** | **69** | **PD** |
| **Labiatae** | **Elsholtzia flava (Benth.) Benth.** | **herb** | **14** | **14** | **21** | **21** | **22** | **28** | **PD** |
| **Labiatae** | **Gomphostemma microdon Dunn** | **tree** | **0** | **0** | **/** | **50** | **49** | **/** | **PD** |
| **Labiatae** | **Leucas martinicensis (Jacq.) R. Br.** | **tree** | **0** | **0** | **/** | **35** | **29** | **/** | **PD** |
| **Labiatae** | **Leonurus artemisia (Lour.) S. Y. Hu** | **herb** | **0** | **0** | **0** | **0** | **0** | **0** | **PD** |
| **Labiatae** | **Perilla frutescens (L.) Britt.** | **herb** | **0** | **0** | **0** | **79** | **81** | **87** | **PD** |
| **Lardizabalaceae** | **Stauntonia brunoniana Wall. ex Hems.** | **vine** | **0** | **0** | **0** | **0** | **0** | **0** | **MPD** |
| **Lauraceae** | **Actinodaphne henryi Gamble** | **tree** | **0** | **0** | **0** | **0** | **0** | **0** | **PD** |
| **Lauraceae** | **Cinnamomum burmannii (C. G. et Th. Nees) Bl.** | **tree** | **0** | **0** | **0** | **41** | **49** | **32** | **PD** |
| **Lauraceae** | **Cinnamomum glanduliferum (Wall.) Nees** | **tree** | **0** | **0** | **0** | **0** | **0** | **0** | **PD** |
| **Lauraceae** | **Cinnamomum porrectum (Roxb.) Kosterm** | **tree** | **0** | **0** | **0** | **0** | **0** | **0** | **PD** |
| **Lauraceae** | **Cryptocarya acutifolia H. W. Li** | **tree** | **0** | **0** | **13** | **87** | **60** | **13** | **PD** |
| **Lauraceae** | **Litsea elongata (Wall. ex Nees) Benth. et Hook. f.** | **tree** | **7** | **2** | **0** | **15** | **18** | **10** | **PD** |
| **Lauraceae** | **Litsea euosma W.W.Sm.** | **tree** | **0** | **0** | **0** | **0** | **0** | **0** | **PD** |
| **Lauraceae** | **Lindera glauca (Sieb. et Zucc.) Bl.** | **shrub** | **0** | **0** | **0** | **0** | **0** | **0** | **PD** |
| **Lauraceae** | **Litsea glutinosa. (Lour.) C.B. Rob.** | **tree** | **0** | **0** | **0** | **0** | **5** | **5** | **PD** |
| **Lauraceae** | **Litsea pierrei Lec. var. szemois Liou** | **tree** | **0** | **0** | **0** | **0** | **3** | **23** | **PD** |
| **Lauraceae** | **Litsea pungens Hemsl.** | **tree** | **0** | **0** | **0** | **0** | **0** | **27** | **PD** |
| **Lauraceae** | **Litsea umbellata (Lour.) Merr.** | **tree** | **0** | **0** | **0** | **0** | **0** | **0** | **PD** |
| **Lauraceae** | **Machilus rufipes H. W. Li** | **tree** | **0** | **0** | **0** | **0** | **0** | **0** | **PD** |
| **Lauraceae** | **Pharbitis purpurea (L.) Voigt** | **tree** | **0** | **0** | **0** | **43** | **63** | **17** | **PD** |
| **Lauraceae** | **Phoebe lanceolata (Nees) Nees** | **tree** | **0** | **0** | **0** | **3** | **0** | **67** | **PD** |
| **Loganiaceae** | **Gelsemium elegans (Gardn. et Champ.) Benth.** | **vine** | **0** | **0** | **0** | **0** | **77** | **77** | **PD** |
| **Malpighiaceae** | **Aspidopterys floribunda Hutch.** | **vine** | **60** | **56** | **56** | **62** | **64** | **62** | **PD** |
| **Magnoliaceae** | **Paramichelia baillonii (Pierre) Hu** | **tree** | **0** | **0** | **2** | **5** | **3** | **17** | **MPD** |
| **Malvaceae** | **Abelmoschus muliensis Feng** | **shrub** | **3** | **2** | **1** | **16** | **12** | **13** | **PY** |
| **Malvaceae** | **Hibiscus mutabilis L.** | **herb** | **9** | **12** | **12** | **30** | **23** | **21** | **PY** |
| **Malvaceae** | **Sida acuta Burm. f.** | **shrub** | **2** | **1** | **1** | **3** | **12** | **6** | **PY** |
| **Malvaceae** | **Sida szechuensis Mast.** | **shrub** | **3** | **3** | **4** | **11** | **7** | **7** | **PY** |
| **Malvaceae** | **Sida sp.** | **shrub** | **5** | **0** | **5** | **5** | **5** | **3** | **PY** |
| **Marantaceae** | **Phrynium capitatum Willd.** | **herb** | **11** | **15** | **24** | **52** | **32** | **37** | **PD** |
| **Melastomataceae** | **Blastus cochinchinensis Lour.** | **tree** | **0** | **0** | **0** | **0** | **0** | **0** | **PD** |
| **Melastomataceae** | **Clerodendrum bungei Steud.** | **shrub** | **0** | **0** | **0** | **0** | **35** | **49** | **PD** |
| **Melastomataceae** | **Melastoma affine D. Don** | **tree** | **0** | **0** | **0** | **80** | **0** | **80** | **PD** |
| **Melastomataceae** | **Osbeckia crinita Benth. ex C. B. Clarke** | **shrub** | **83** | **84** | **83** | **84** | **100** | **100** | **ND** |
| **Melastomataceae** | **Sarcopyramis bodinieri var. delicata (C. B. Robins.) C. Chen** | **herb** | **0** | **0** | **0** | **95** | **80** | **0** | **PD** |
| **Meliaceae** | **Amoora tetrapetala (Pierre) Pellegr. var. macrophylla (H.L.Li)C.Y.Wu** | **tree** | **0** | **0** | **0** | **0** | **0** | **0** | **PD** |
| **Meliaceae** | **Aphanamixis polystachya (Wall.) R. N. Parker** | **tree** | **50** | **50** | **57** | **57** | **73** | **73** | **ND** |
| **Meliaceae** | **Cipadessa baccifera (Roth) Miq.** | **shrub** | **0** | **0** | **17** | **23** | **51** | **67** | **PD** |
| **Meliaceae** | **Dysoxylum binectariferum (Roxb.) Hook. f. ex Bedd.** | **tree** | **0** | **0** | **0** | **0** | **0** | **0** | **PD** |
| **Meliaceae** | **Toona ciliata Roem.** | **tree** | **0** | **0** | **0** | **0** | **0** | **0** | **PD** |
| **Meliaceae** | **Walsura yunnanensis C. Y. Wu** | **tree** | **53** | **47** | **47** | **60** | **63** | **53** | **ND** |
| **Menispermaceae** | **Pericampylus glaucus (Lam.) Merr.** | **vine** | **0** | **0** | **0** | **0** | **0** | **3** | **PD** |
| **Moraceae** | **Artocarpus hypargyreus Hance** | **tree** | **0** | **0** | **0** | **19** | **18** | **28** | **PD** |
| **Moraceae** | **Ficus stenophylla Hemsl.** | **shrub** | **0** | **71** | **79** | **0** | **74** | **81** | **ND** |
| **Moraceae** | **Ficus semicordata Buch.-Ham.exJ.E.Smith** | **tree** | **81** | **86** | **88** | **89** | **87** | **88** | **ND** |
| **Moraceae** | **Ficus altissima Bl.** | **tree** | **83** | **95** | **100** | **100** | **95** | **100** | **ND** |
| **Moraceae** | **Ficus auriculata Lour.** | **tree** | **95** | **97** | **97** | **95** | **97** | **97** | **ND** |
| **Moraceae** | **Ficus benjamina L.** | **tree** | **83** | **77** | **78** | **97** | **84** | **95** | **ND** |
| **Moraceae** | **Ficus cyrtophylla Wall. ex Miq.** | **tree** | **0** | **0** | **0** | **0** | **0** | **0** | **PD** |
| **Moraceae** | **Ficus fistulosa Reinw.** | **tree** | **75** | **90** | **89** | **93** | **91** | **91** | **ND** |
| **Moraceae** | **Ficus hispida L.** | **tree** | **0** | **0** | **0** | **0** | **0** | **0** | **PD** |
| **Moraceae** | **Ficus hispida L. f. var. badiostrigosa Corner** | **tree** | **33** | **58** | **31** | **73** | **72** | **69** | **PD** |
| **Moraceae** | **Ficus ischnopoda Mig.** | **tree** | **0** | **0** | **0** | **0** | **0** | **0** | **PD** |
| **Moraceae** | **Ficus maclellandi King var. rhododendrifolia Corner** | **tree** | **77** | **77** | **88** | **89** | **96** | **98** | **ND** |
| **Moraceae** | **Ficus oligodon Miq.** | **tree** | **100** | **100** | **100** | **100** | **100** | **100** | **ND** |
| **Moraceae** | **Ficus pubigera (Wall. ex Miq.) Miq.** | **vine** | **99** | **99** | **99** | **100** | **100** | **99** | **ND** |
| **Moraceae** | **Ficus racemosa L.** | **tree** | **77** | **61** | **61** | **92** | **92** | **77** | **ND** |
| **Moraceae** | **Ficus religiosa L.** | **tree** | **82** | **63** | **71** | **83** | **87** | **77** | **ND** |
| **Moraceae** | **Ficus semicordata Buch.-Ham. exJ.E.Smith** | **tree** | **82** | **83** | **87** | **92** | **89** | **91** | **ND** |
| **Moraceae** | **Ficus sp.** | **tree** | **89** | **95** | **89** | **95** | **97** | **98** | **ND** |
| **Moraceae** | **Streblus indicus (Bureau) Corner** | **tree** | **22** | **16** | **16** | **29** | **29** | **22** | **PD** |
| **Musaceae** | **Musa acuminata Colla** | **shrub** | **85** | **55** | **55** | **85** | **85** | **85** | **MD** |
| **Musaceae** | **Musa sp.** | **tree** | **0** | **0** | **0** | **0** | **0** | **0** | **MPD** |
| **Myristicaceae** | **Knema globularia (Lam.) Warb.** | **tree** | **0** | **0** | **0** | **83** | **87** | **93** | **MPD** |
| **Myristicaceae** | **Myristica yunnanensis Y. H. Li** | **tree** | **0** | **0** | **5** | **10** | **15** | **7** | **MPD** |
| **Myristicaceae** | **Knema erratica (Hook. f. et Thoms.) J. Sincl.** | **tree** | **0** | **0** | **/** | **80** | **80** | **/** | **MPD** |
| **Myrsinaceae** | **Ardisia japonica (Thunb.) Blume** | **shrub** | **0** | **0** | **0** | **50** | **69** | **87** | **PD** |
| **Myrsinaceae** | **Ardisia neriifolia Wall** | **shrub** | **43** | **31** | **31** | **50** | **43** | **50** | **PD** |
| **Myrsinaceae** | **Embelia floribunda Wall.** | **vine** | **0** | **0** | **0** | **0** | **0** | **0** | **PD** |
| **Myrsinaceae** | **Embelia ribes Burm. f.** | **vine** | **0** | **0** | **0** | **0** | **0** | **7** | **PD** |
| **Myrsinaceae** | **Embelia sessiliflora Kurz** | **vine** | **0** | **0** | **0** | **0** | **0** | **0** | **PD** |
| **Myrsinaceae** | **Embelia sp.** | **vine** | **3** | **3** | **3** | **52** | **37** | **24** | **PD** |
| **Myrsinaceae** | **Maesa indica (Roxb.) A. DC.** | **shrub** | **42** | **11** | **10** | **87** | **73** | **71** | **PD** |
| **Myrsinaceae** | **Maesa japonica (Thunb.) Moritzi.** | **shrub** | **0** | **0** | **0** | **93** | **98** | **4** | **PD** |
| **Myrsinaceae** | **Myrsine seguinii H. Lév.** | **tree** | **0** | **0** | **0** | **87** | **0** | **0** | **PD** |
| **Myrsinaceae** | **Myrsine semiserrata Wall.** | **shrub** | **71** | **82** | **83** | **98** | **83** | **88** | **ND** |
| **Myrtaceae** | **Cleistocalyx operculatus (Roxb.) Merr.** | **tree** | **60** | **65** | **60** | **75** | **75** | **65** | **ND** |
| **Myrtaceae** | **Decaspermum fruticosum J. R. et G. Forst.** | **tree** | **0** | **0** | **0** | **0** | **8** | **11** | **PD** |
| **Nyssaceae** | **Camptotheca acuminata Decne.** | **tree** | **31** | **16** | **7** | **58** | **44** | **47** | **PD** |
| **Olacaceae** | **Erythropalum scandens Bl.** | **vine** | **0** | **0** | **0** | **0** | **0** | **0** | **MPD** |
| **Oleaceae** | **Fraxinus floribundus Wall.** | **tree** | **34** | **14** | **19** | **36** | **37** | **29** | **PD** |
| **Oleaceae** | **Jasminum coarctatum Roxb.** | **shrub** | **0** | **0** | **0** | **0** | **0** | **0** | **PD** |
| **Onagraceae** | **Ludwigia octovalvis(Jacq.) Raven** | **herb** | **2** | **2** | **25** | **41** | **37** | **58** | **PD** |
| **Passifloraceae** | **Adenia cardiophylla (Mast.) Engl.** | **vine** | **0** | **0** | **0** | **0** | **0** | **0** | **PD** |
| **Piperaceae** | **Piper szemaoense C. DC.** | **vine** | **0** | **0** | **0** | **11** | **12** | **0** | **PD** |
| **Pittosporaceae** | **Pittosporum crispulum Gagnep.** | **tree** | **0** | **0** | **0** | **35** | **0** | **0** | **MPD** |
| **Pittosporaceae** | **Pittosporum paniculiferum Chang et Yan** | **tree** | **0** | **0** | **0** | **0** | **14** | **14** | **MPD** |
| **Pittosporaceae** | **Pittosporum tobira (Thunb.) Ait.** | **tree** | **0** | **0** | **0** | **5** | **0** | **3** | **MPD** |
| **Polygalaceae** | **Polygala arillata Buch.-Ham. ex D. Don** | **shrub** | **0** | **0** | **0** | **0** | **0** | **3** | **PD** |
| **Polygonaceae** | **Polygonum hydropiper L.** | **herb** | **2** | **3** | **2** | **15** | **85** | **26** | **PD** |
| **Polygonaceae** | **Polygonum molle D. Don** | **herb** | **0** | **0** | **0** | **24** | **36** | **31** | **PD** |
| **Polygonaceae** | **Polygonum molle D. Don var. rude (Meisn.) A.J.Li** | **herb** | **/** | **0** | **0** | **/** | **37** | **32** | **PD** |
| **Polygonaceae** | **Polygonum sp.** | **herb** | **6** | **3** | **2** | **11** | **37** | **3** | **PD** |
| **Rhamnaceae** | **Berchemia hirtella Tsai et Feng** | **vine** | **0** | **0** | **0** | **0** | **0** | **0** | **PD** |
| **Rhamnaceae** | **Hovenia acerba Lindl. var. kiukiangensis (Hu et Cheng) C.Y.Wu ex Y.L.Chen** | **tree** | **0** | **13** | **0** | **0** | **15** | **26** | **PD** |
| **Rhamnaceae** | **Rhamnus napalensis (Wall.) Laws.** | **vine** | **0** | **0** | **0** | **0** | **21** | **23** | **PD** |
| **Rhizophoraceae** | **Carallia brachiata (Lour.) Merr.** | **tree** | **22** | **16** | **51** | **78** | **64** | **64** | **PD** |
| **Rhizophoraceae** | **Carallia sp.** | **tree** | **0** | **0** | **0** | **0** | **0** | **0** | **PD** |
| **Rosaceae** | **Agrimonia pilosa Ldb. var. nepalensis (D.Don) Nakai** | **herb** | **9** | **0** | **0** | **26** | **36** | **1** | **PD** |
| **Rosaceae** | **Docynia indica (Wall.) Dcne.** | **tree** | **0** | **0** | **0** | **0** | **0** | **0** | **PD** |
| **Rosaceae** | **Pygeum latifolium Miquel var.macrocarpum(T. T. Yü & L. T. Lu) C. Y. Wu & H. Chu** | **tree** | **77** | **83** | **77** | **87** | **100** | **83** | **ND** |
| **Rosaceae** | **Pyrus betulifolia Bunge** | **tree** | **90** | **64** | **64** | **94** | **94** | **90** | **ND** |
| **Rosaceae** | **Rubus alceaefolius Poir.** | **vine** | **0** | **0** | **0** | **0** | **0** | **0** | **PD** |
| **Rosaceae** | **Rubus caudifolius Wuzhi** | **vine** | **0** | **0** | **0** | **0** | **0** | **0** | **PD** |
| **Rubiaceae** | **Aidia cochinchinensis Lour.** | **tree** | **38** | **20** | **22** | **65** | **72** | **60** | **PD** |
| **Rubiaceae** | **Aidia oxyodonta (Drake) Yamazaki** | **shrub** | **0** | **21** | **2** | **37** | **62** | **70** | **PD** |
| **Rubiaceae** | **Brachytome sp.** | **tree** | **0** | **0** | **0** | **0** | **0** | **0** | **PD** |
| **Rubiaceae** | **Brachytome hirtellata Hu** | **shrub** | **0** | **0** | **0** | **25** | **36** | **9** | **PD** |
| **Rubiaceae** | **Brachytome wallichii Hook. f.** | **tree** | **0** | **0** | **0** | **0** | **0** | **0** | **PD** |
| **Rubiaceae** | **Canthium parvifoliam Roxb.** | **tree** | **0** | **0** | **0** | **0** | **0** | **0** | **PD** |
| **Rubiaceae** | **Canthium horridum Bl.** | **tree** | **0** | **0** | **0** | **0** | **0** | **0** | **PD** |
| **Rubiaceae** | **Chassalia curviflora Thwaites** | **shrub** | **0** | **0** | **0** | **0** | **0** | **17** | **PD** |
| **Rubiaceae** | **Diplospora dubia (Lindl.) Masam.** | **tree** | **86** | **84** | **84** | **88** | **87** | **88** | **ND** |
| **Rubiaceae** | **Ixora sp.** | **shrub** | **0** | **0** | **0** | **0** | **10** | **43** | **PD** |
| **Rubiaceae** | **Mussaenda hossei Craib** | **shrub** | **0** | **70** | **0** | **0** | **77** | **0** | **PD** |
| **Rubiaceae** | **Mussaenda macrophylla Wall.** | **tree** | **0** | **0** | **0** | **52** | **35** | **0** | **PD** |
| **Rubiaceae** | **Mussaenda sp.** | **tree** | **0** | **0** | **0** | **0** | **24** | **24** | **PD** |
| **Rubiaceae** | **Neolamarckia cadamba (Roxb.) Bosser** | **tree** | **0** | **0** | **1** | **1** | **2** | **2** | **PD** |
| **Rubiaceae** | **Psychotria calocarpa Kurz** | **shrub** | **0** | **0** | **0** | **9** | **16** | **0** | **MPD** |
| **Rubiaceae** | **Uncaria macrophylla Wall.** | **vine** | **0** | **0** | **0** | **0** | **95** | **95** | **PD** |
| **Rubiaceae** | **Uncaria scandens (Smith) Hutchins.** | **vine** | **0** | **0** | **0** | **77** | **77** | **0** | **PD** |
| **Rubiaceae** | **Uncaria sp.** | **vine** | **0** | **0** | **0** | **57** | **65** | **68** | **PD** |
| **Rutaceae** | **Evodia fraxinifolia (D. Don) Hook.** | **tree** | **0** | **0** | **0** | **0** | **0** | **0** | **PD** |
| **Rutaceae** | **Micromelum integerrimum (Buch.-Ham.) Roem. var. mollissimum Tanaka** | **shrub** | **0** | **0** | **0** | **0** | **0** | **0** | **PD** |
| **Rutaceae** | **Micromelum integerrimum (Buch.-Ham.) Roem.** | **tree** | **90** | **90** | **93** | **93** | **90** | **93** | **ND** |
| **Rutaceae** | **Toddalia asiatica (L.) Lam.** | **vine** | **0** | **0** | **0** | **0** | **0** | **0** | **PD** |
| **Rutaceae** | **Toddalia sp.** | **vine** | **0** | **0** | **0** | **0** | **0** | **0** | **PD** |
| **Sabiaceae** | **Meliosma simplicifolia (Roxb.) Walp.** | **tree** | **0** | **0** | **0** | **0** | **3** | **15** | **PD** |
| **Santalaceae** | **Osyris quadripartita Salzm. ex Decne.** | **shrub** | **0** | **0** | **0** | **0** | **1** | **0** | **MPD** |
| **Santalaceae** | **Scleropyrum wallichianum (Wight et Arn.) Arn. var. mekongense (Gagnep.) Lecomte** | **tree** | **0** | **0** | **0** | **0** | **0** | **0** | **MPD** |
| **Sapindaceae** | **Aphania rubra (Roxb.) Radlk.** | **shrub** | **0** | **0** | **0** | **97** | **97** | **75** | **PY** |
| **Sapindaceae** | **Dodonaea viscosa (L.) Jacq.** | **tree** | **0** | **0** | **0** | **0** | **3** | **3** | **PY** |
| **Sapindaceae** | **Nephelium chryseum Bl.** | **tree** | **43** | **43** | **60** | **97** | **97** | **60** | **ND** |
| **Sapindaceae** | **Sapindus mukorossi Gaertn.** | **tree** | **89** | **71** | **71** | **91** | **89** | **89** | **ND** |
| **Sapotaceae** | **Madhuca pasquieri (Dubard) H. J. Lam** | **tree** | **57** | **57** | **57** | **67** | **83** | **57** | **PD** |
| **Sapotaceae** | **Pouteria grandifolia (Wall.) Baehni** | **tree** | **0** | **0** | **0** | **17** | **0** | **0** | **PD** |
| **Sapotaceae** | **Sarcosperma griffithii Hook. f. ex C. B. Clarke** | **tree** | **0** | **0** | **0** | **0** | **0** | **0** | **PD** |
| **Sapotaceae** | **Sarcosperma kachinense (King et Prain) Exell. var. simondii (Gagn.) Lam et P.Royen** | **tree** | **0** | **0** | **0** | **0** | **0** | **0** | **PD** |
| **Sapotaceae** | **Sarcosperma laurinum (Benth.) Hook. f.** | **tree** | **10** | **7** | **7** | **87** | **93** | **40** | **PD** |
| **Schizandraceae** | **Subgen. Maximowiczia (Rupr.) Law** | **vine** | **0** | **0** | **0** | **0** | **0** | **0** | **MPD** |
| **Simarubaceae** | **Brucea javanica (L.) Merr.** | **shrub** | **0** | **0** | **0** | **0** | **0** | **0** | **PD** |
| **Smilacaceae** | **Smilax china L.** | **vine** | **0** | **0** | **0** | **0** | **51** | **42** | **MPD** |
| **Solanaceae** | **Solanum coagulans Forsk.** | **shrub** | **0** | **4** | **0** | **1** | **4** | **9** | **PD** |
| **Solanaceae** | **Solanum indicum L.** | **shrub** | **3** | **4** | **3** | **23** | **37** | **5** | **PD** |
| **Solanaceae** | **Solanum nigrum L.** | **herb** | **0** | **0** | **0** | **1** | **1** | **0** | **PD** |
| **Solanaceae** | **Solanum spirale Roxb.** | **shrub** | **8** | **0** | **0** | **11** | **11** | **8** | **PD** |
| **Solanaceae** | **Solanum torvum Swartz** | **shrub** | **0** | **0** | **0** | **0** | **0** | **1** | **PD** |
| **Staphyleaceae** | **Turpinia cochinchinensis (Lour.) Merr.** | **tree** | **0** | **0** | **0** | **1** | **0** | **3** | **PD** |
| **Sterculiaceae** | **Abroma angusta（L.）L.f.** | **shrub** | **0** | **5** | **2** | **6** | **11** | **11** | **PY** |
| **Sterculiaceae** | **Helicteres angustifolia L.** | **shrub** | **0** | **0** | **1** | **7** | **3** | **11** | **PY** |
| **Sterculiaceae** | **Pterygota alata(Roxb.)R.Br** | **tree** | **0** | **0** | **0** | **0** | **0** | **0** | **PY** |
| **Taccaceae** | **Tacca chantrieri Andre** | **herb** | **0** | **0** | **0** | **89** | **91** | **96** | **PD** |
| **Theaceae** | **Anneslea fragrans Wall.** | **tree** | **0** | **0** | **0** | **0** | **0** | **0** | **PD** |
| **Theaceae** | **Pyrenaria sp.** | **tree** | **13** | **15** | **27** | **27** | **31** | **31** | **PD** |
| **Thymelaeaceae** | **Wikstroemia indica C. A. Mey** | **shrub** | **1** | **0** | **1** | **31** | **39** | **0** | **PD** |
| **Tiliaceae** | **Corchorus aestuans L. var. aestuans** | **tree** | **0** | **0** | **0** | **0** | **2** | **2** | **PY** |
| **Tiliaceae** | **Microcos paniculata L.** | **tree** | **0** | **0** | **0** | **0** | **0** | **0** | **PY** |
| **Ulmaceae** | **Celtis timorensis Spanoghe** | **tree** | **1** | **3** | **1** | **15** | **17** | **8** | **PD** |
| **Ulmaceae** | **Ulmus lanceaefolia Roxb.** | **tree** | **82** | **76** | **76** | **87** | **82** | **87** | **ND** |
| **Umbelliferae** | **Hydrocotyle nepalensis Hook.** | **tree** | **0** | **0** | **0** | **0** | **0** | **0** | **MPD** |
| **Umbelliferae** | **Hydrocotyle sibthorpioides Lam.** | **tree** | **0** | **0** | **0** | **0** | **0** | **0** | **MPD** |
| **Urticaceae** | **Boehmeria platyphylla D. Don var.scabrella(Roxb.)** | **shrub** | **69** | **62** | **66** | **69** | **76** | **67** | **ND** |
| **Urticaceae** | **Debregeasia longifolia (Burm. F.) Wedd.** | **tree** | **0** | **0** | **0** | **0** | **0** | **0** | **PD** |
| **Urticaceae** | **Oreocnide integrifolia (Gaudich.) Miq.** | **tree** | **0** | **0** | **0** | **87** | **83** | **0** | **PD** |
| **Urticaceae** | **Pilea notata C. H. Wright** | **herb** | **0** | **17** | **9** | **31** | **32** | **35** | **PD** |
| **Urticaceae** | **Poikilospermum suaveolens (Bl.) Merr.** | **tree** | **0** | **0** | **0** | **0** | **0** | **0** | **PD** |
| **Verbenaceae** | **Callicarpa arborea Roxb.** | **tree** | **0** | **0** | **0** | **25** | **18** | **0** | **PD** |
| **Verbenaceae** | **Callicarpa bodinieri Levl.** | **shrub** | **1** | **94** | **12** | **90** | **94** | **85** | **PD** |
| **Verbenaceae** | **Callicarpa giraldii Hesse ex Rehd.** | **shrub** | **5** | **17** | **7** | **9** | **23** | **36** | **PD** |
| **Verbenaceae** | **Callicarpa rubella Lindl.** | **shrub** | **0** | **0** | **0** | **73** | **41** | **92** | **PD** |
| **Verbenaceae** | **Callicarpa rubella Lindl. var. rubella f. angustata P'ei** | **tree** | **0** | **/** | **0** | **69** | ***/*** | **54** | **PD** |
| **Verbenaceae** | **Clerodendrum philippinum Schauer var. simplex Moldenke** | **shrub** | **0** | **0** | **0** | **0** | **0** | **0** | **PD** |
| **Verbenaceae** | **Vitex trifolia L.** | **shrub** | **0** | **0** | **0** | **0** | **3** | **3** | **PD** |
| **Vitaceae** | **Cayratia japonica (Thunb.) Gagnep.** | **herb** | **0** | **0** | **0** | **0** | **0** | **0** | **MPD** |
| **Vitaceae** | **Cissus repens Lamk.** | **vine** | **0** | **0** | **0** | **0** | **0** | **0** | **MPD** |
| **Vitaceae** | **Cissus javana DC.** | **vine** | **0** | **0** | **1** | **17** | **10** | **27** | **MPD** |
| **Vitaceae** | **Leea indica (Burm. F.) Merr.** | **shrub** | **1** | **1** | **3** | **27** | **90** | **97** | **MPD** |
| **Vitaceae** | **Tetrastigma lenticellatum C. Y. Wu ex W. T. Wang** | **vine** | **0** | **7** | **0** | **11** | **7** | **5** | **MPD** |
| **Vitaceae** | **Tetrastigma obtectum (Wall.) Planch.** | **vine** | **0** | **0** | **0** | **0** | **0** | **0** | **MPD** |
| **Vitaceae** | **Tetrastigma obovatum (Laws.) Gagnep.** | **vine** | **0** | **16** | **20** | **51** | **22** | **22** | **MPD** |
| **Vitaceae** | **Tetrastigma obtectum (Wall.) Planch.** | **vine** | **0** | **0** | **0** | **0** | **5** | **5** | **MPD** |
| **Vitaceae** | **Tetrastigma planicaule (Hook.) Gagnep.** | **vine** | **0** | **0** | **0** | **0** | **3** | **3** | **MPD** |
| **Vitaceae** | **Tetrastigma sp.** | **vine** | **0** | **36** | **7** | **43** | **77** | **63** | **MPD** |
| **Vitaceae** | **Vitis adstricta Hance** | **vine** | **0** | **2** | **2** | **0** | **2** | **2** | **MPD** |
| **Zingiberaceae** | **Alpinia blepharocalyx K.Schum.** | **herb** | **0** | **0** | **0** | **2** | **1** | **7** | **PD** |
| **Zingiberaceae** | **Amomum maxinum Roxb.** | **herb** | **0** | **0** | **0** | **0** | **0** | **0** | **PD** |
| **Zingiberaceae** | **Amomum tsao-ko Crevost** | **herb** | **0** | **0** | **0** | **0** | **0** | **0** | **PD** |
| **Zingiberaceae** | **Costus speciosus (Koen.) Smith** | **herb** | **5** | **5** | **7** | **7** | **13** | **13** | **PD** |
| **Zingiberaceae** | **Costus sp.** | **herb** | **0** | **0** | **0** | **8** | **9** | **4** | **PD** |
| **Zingiberaceae** | **Costus sp.** | **herb** | **0** | **39** | **21** | **38** | **40** | **48** | **PD** |
| **Zingiberaceae** | **Hedychium forrestii Diels** | **herb** | **19** | **6** | **8** | **19** | **50** | **14** | **PD** |
| **Zingiberaceae** | **Zinger xishangbannaense S. Q. Tong** | **herb** | **96** | **68** | **92** | **98** | **96** | **98** | **ND** |
